# Single isocenter versus dual isocenter treatment using flattening filter‐free and jaw‐tracking volumetrically modulated arc therapy for boot‐shaped lung cancer: Evaluation of dosimetric and feasibility

**DOI:** 10.1002/acm2.14292

**Published:** 2024-01-29

**Authors:** Lei Zhang, Hang Cheng, Fenglei Du, Kainan Shao, Shiming Zheng, Yiwei Yang, Guoping Shan

**Affiliations:** ^1^ Department of Radiation Physics Zhejiang Cancer Hospital Hangzhou Zhejiang China; ^2^ Hangzhou Institute of Medicine(HIM) Chinese Academy of Sciences Hangzhou Zhejiang China; ^3^ Radiotherapy Technology Department Yuyao People's Hospital of Zhejiang Province NingBo Zhejiang China

**Keywords:** boot‐shaped lung cancer, dual‐isocenter, jaw tracking VMAT, radiation pneumonitis, radiotherpy dosage

## Abstract

**Background:**

To determine whether a dual‐isocenter volumetrically modulated arc therapy (VMAT) technique results in lower normal pulmonary dosage compared to a traditional single isocenter technique for boot‐shaped lung cancer.

**Methods:**

A cohort of 15 patients with advanced peripheral or central lung cancer who had metastases in the mediastinum and supraclavicular lymph nodes was randomly selected for this retrospective study. VMAT plans were generated for each patient using two different beam alignment techniques with the 6‐MV flattening filter‐free (FFF) photon beam: single‐isocenter jaw‐tracking VMAT based on the Varian TrueBeam linear accelerator (S‐TV), and dual‐isocenter VMAT based on both TrueBeam (D‐TV) and Halcyon linear accelerator (D‐HV). For all 45 treatment plans, planning target volume (PTV) dose coverage, conformity/homogeneity index (CI/HI), mean heart dose (MHD), mean lung dose (MLD) and the total lung tissue receiving 5, 20, 30 Gy (V_5_, V_20_, V_30_) were evaluated. The monitor units (MUs), delivery time, and plan quality assurance (QA) results were recorded.

**Results:**

The quality of the objectives of the three plans was comparable to each other. In comparison with S‐TV, D‐TV and D‐HV improved the CI and HI of the PTV (*p* < 0.05). The MLD was 13.84 ± 1.44 Gy (mean ± SD) for D‐TV, 14.22 ± 1.30 Gy and 14.16 ± 1.42 Gy for S‐TV and D‐HV, respectively. Lungs‐V_5Gy_ was 50.78 ± 6.24%, 52.00 ± 7.32% and 53.36 ± 8.48%, Lungs‐V_20Gy_ was 23.72 ± 2.27%, 26.18 ± 2.86% and 24.96 ± 3.09%, Lungs‐V30Gy was 15.69 ± 1.76%, 17.20 ± 1.72% and 16.52 ± 2.07%. Compared to S‐TV, D‐TV provided statistically significant better protection for the total lung, with the exception of the lungs‐V_5_. All plans passed QA according the gamma criteria of 3%/3 mm.

**Conclusions:**

Taking into account the dosimetric results and published clinical data on radiation‐induced pulmonary injury, dual‐isocenter jaw‐tracking VMAT may be the optimal choice for treating boot‐shaped lung cancer.

## 1 BACKGROUND

Lung cancer is a leading cause of cancer‐related deaths worldwide.^1^ In China, the incidence and mortality rates of lung cancer account for more than one third of the world's total.^2^ Radical chemoradiotherapy is the standard of care for lung patients with locally advanced or distant lymph node metastases who are not candidates for, or decline, surgical intervention.^3^, ^4^ In medium‐term and advanced peripheral and central lung cancer, the target area for radiotherapy is typically large due to the higher risk of metastases in the mediastinum and supraclavicular lymph nodes. The target resembles a boot in coronal view, hence the term “boot‐shaped”. A potential challenge in designing a radiotherapy treatment plan for lung cancer with a sizable longitudinal tumor is to ensure sufficient tumor coverage while reducing the dose to normal lung tissue. Reducing the dosage to the normal lung could potentially decrease the risk of patients developing radiation pneumonitis (RP). For the treatment of lung cancer, intensity modulated radiation therapy (IMRT) techniques offer a significant dosimetric and clinical advantage.^5^, ^6^, ^7^, ^8^, ^9^ Research has demonstrated that employing IMRT as a treatment for lung cancer results in minimal rates of pulmonary and esophageal toxicity, as well as positive clinical outcomes in terms of patient survival.^5^ Volume Modulated Arc Therapy (VMAT) is a novel IMRT method that utilizes a rotating gantry and continuous beam modulation to deliver radiation doses to a tumor, and several studies suggested that VMAT could be an effective and safe treatment option.^10^, ^11^, ^12^, ^13^ Zhang et al.^12^ conducted a study comparing the efficacy of VMAT and IMRT techniques for the treatment of non‐small cell lung cancer and indicated that the VMAT group significantly reduced heart‐V_20_, V_30_ and V_40_, but lungs‐V_5_ and V_10_ were higher than those in the IMRT. Abbas et al.^13^ investigated the benefit of VMAT for the large planning target volume (PTV) in esophageal cancer. Meanwhile, radiotherapy technology has continued to evolve. Recently, several reports have examined various VMAT techniques for treating lung cancer, analyzing their impact on normal lung dose and confirming their feasibility.^14^, ^15^, ^16^ One of those studies investigated the benefit of the fixed jaw and jaw tracking VMAT technique, one study evaluated the benefit of the flattened filter‐free photon beam, and one study added a non‐coplanar arc to reduce dose to organs at risk (OAR). Nevertheless, to our knowledge, nearly none of the studies investigated the dosimetric effect of dual‐isocenter jaw‐tracking VMAT in lung cancer with a large longitudinal tumor target. The potential reduction in dose spillover to the normal lung using dual‐isocenter beam alignment technique was confirmed in multi‐lesion lung stereotactic body radiotherapy (SBRT)^17^ and synchronous bilateral breast VMAT.^18^, ^19^ In addition, due to the limited field size of Halcyon linear accelerator (28 cm × 28 cm) , some large tumor targets may also require dual isocenter planning to achieve sufficient target coverage. Therefore, the purpose of this study was to assess whether the use of a dual‐isocenter FFF VMAT technique for the treatment of boot‐shaped lung tumors can result in a reduced normal lung dose compared to the traditional single‐isocenter technique.

## 2 METHODS

### 2.1 Patients characteristic

This study included a subset of 15 patients with boot‐shaped advanced lung cancer who underwent radiotherapy at Zhejiang Cancer Hospital (Hangzhou, China) and was conducted between December 2020 and November 2022. The patients were diagnosed with lung cancer and were found to have metastatic lymph nodes in both the mediastinum and supraclavicular regions. All patients were staged using American Joint Committee on Cancer (AJCC) 7th edition staging system. Ethical approval for this low‐risk study has been obtained from the local Ethics Committee.

### 2.2 Delineation of target and organs at risk

All patients were scanned in the head‐first supine position, immobilized with a thermoplastic mask. Philips BrillianceTM Big Bore CT (Philips, Netherlands) was used for a 5 mm thick CT scan. All

CT images were transferred via the network to the RayStation 9.0 treatment planning system (RaySearch, Sweden) for the purpose of treatment planning. Subspecialist radiation oncologists (RO) delineated targets and OARs according to ESTRO consensus guidelines,^20^, ^21^ which were then reviewed by the supervising RO. Gross tumor volume (GTV) was delineated on the planning CT combined with contrast‐enhanced CT, PET/CT and pathology reports. GTV‐T included the visualization of pulmonary lesions and GTV‐N included metastatic lymph nodes in the mediastinum as well as supraclavicular lymph nodes. A 0.6 cm expansion beyond the GTV was added to form the clinical target volume (CTV). To account for setup errors and respiratory movement, the PTV was obtained by expanding the clinical target volume (CTV) isotropically by 0.5 cm. The defined OARs consisted of the total lung, heart, spinal cord and esophagus. Total lung was defined as the sum of the right and left lung volumes, minus the GTV.

### 2.3 Treatment planning

Three FFF VMAT plans were generated for each case: S‐TV, D‐TV, D‐HV. All treatment plans were designed in the RayStation 9.0 TPS using direct machine parameter optimization (DMPO) algorithm with a grid resolution of 2.5 mm. Varian TrueBeam linear accelerator (Varian, USA) with 6 MV FFF photon beam at a maximum dose rate of 1400MU/min was used for S‐TV and D‐TV plans while the jaw‐tracking option was enabled. Varian Halycon linear accelerator (Varian, USA) with 6 MV FFF photon beam of 800MU/min was adapted for D‐HV plans.

**FIGURE 1:**
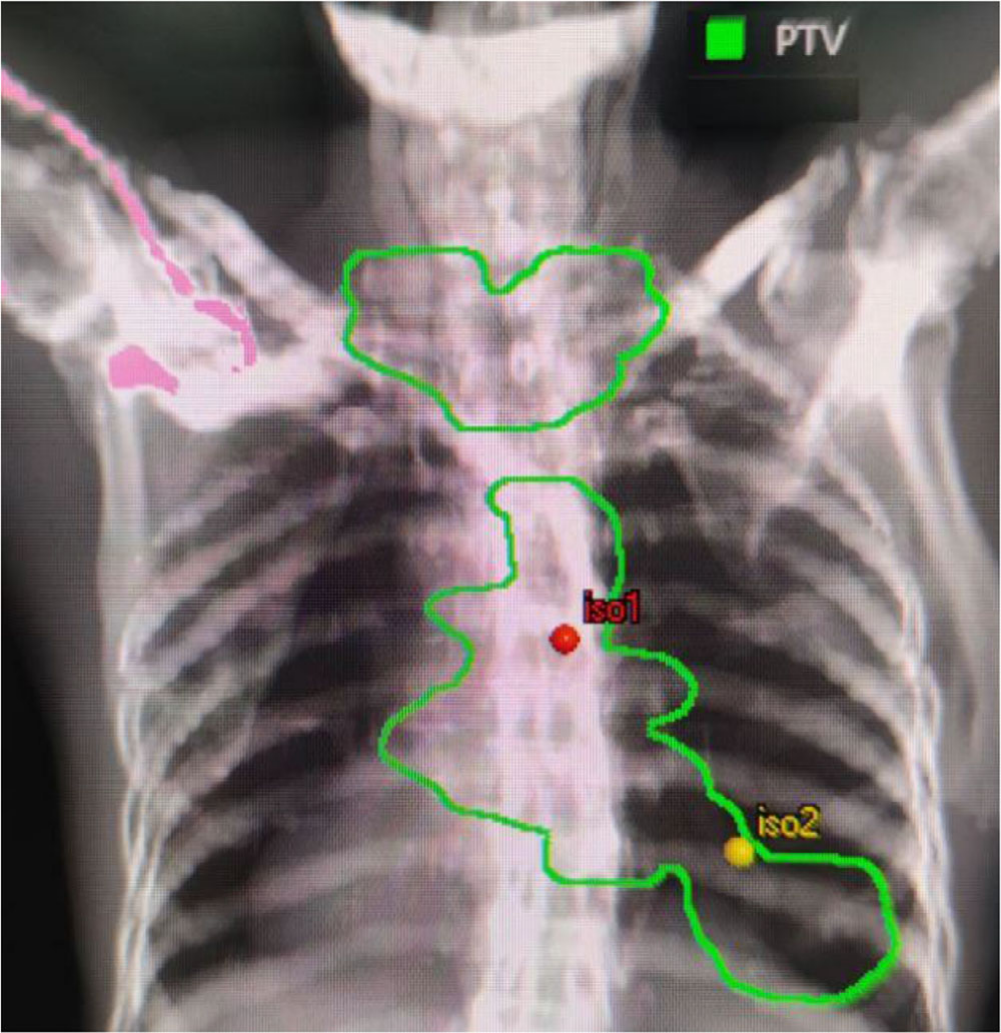
Dual‐isocenter of the plans.

For the dual‐isocenter D‐TV and D‐HV plans, one isocenter was placed in the center of the upper (upper and middle tumor, iso1) and the other in the center of the sole (lung tumor, iso2), as shown in Figure 1. For iso1, the beam angles were set to 182°−225° (Clockwise, counter‐clockwise), 310°−50° (CW, CCW), and 135°−178° (CW, CCW). Depending on the patient's actual situation, the beam angle can be modulated slightly. The collimator angles were set at 15° and 345° to minimize the opening of the MLC between the tumors. For iso2, an additional 2 coplanar half arcs were used. All beams were algorithmically optimized in the same plan.

For the single‐isocenter VMAT, S‐TV treatment plans were designed using a single isocenter placed at the geometric center of the PTV. In order to compare with dual‐isocenter plans, the beam angles and collimator angles were the same as dual‐isocenter plans.

### 2.4 Treatment planning evaluation

A prescription dose of 60 Gy delivered in 30 fractions was given to the PTV for each patient. To allow a direct comparison with three VMAT techniques, the dose was normalized to ensure that at least 95% of the PTV volume received 100% of the prescribed dose(V_60Gy_≥95%). Dose to the PTV was evaluated according to the recommendations of the ICRU reporting guideline: D_98%_, D_2%_, D_50%_, Dmean, conformity index (CI), homogeneity index (HI). The calculation of CI and HI was performed using the methodology described in the ICRU Report 83.^22^ If the CI is closer to 1, it indicates a better dose conformity within the PTV. On the other hand, a smaller HI value indicates a more uniform distribution. The assessment objectives of the OARs in this study included mean dose to the lungs and the lungs volume receiving 5, 20, and 30 Gy (V_5_, V_20_, V_30_), mean dose to the heart and the heart volume receiving 20 and 30 Gy (V_20_, V_30_), and the maximum dose to the spinal cord. The optimization objectives and dose‐volume constraints for the three techniques were implemented according to Table 1. Moreover, total monitor units (MUs) were also recorded to assess treatment effectiveness. The Varian on‐board electronic portal imaging device (EPID) was used to assess plan‐specific QA according to the 3 mm/3% analysis standard with a 5% low‐dose threshold.

**TABLE 1:** Patient characteristics, PTV = planning target volume (*n* = 15).

Characteristics	Number of case
Age (years)	
Median [range]	61 [49–74]
Gender	
Male	14
Female	1
PTV width (cm)	
Median [range]	15.0 [13–18.3]
Mean ± SD	15.3 ± 1.6
PTV length (cm)	
Median [range]	22.0 [16.2–30.5]
Mean ± SD	22.5 ± 3.2
PTV volume (cc)	
Median [range]	441.2 [223.8–831.4]
Mean ± SD	446.7 ± 183.5
Total lung volume	
Median [range]	3357.74 [2067.6–4559.5]
Mean ± SD	3354.1 ± 863.9

### 2.5 Statistical analysis

The data collected were analyzed using SPSS 27.0 (IBM Corporation, Armonk, NY, USA) software and presented as “mean ± standard deviation (SD)”. Significant differences between the three treatment planning techniques were tested using Wilcoxon's signed rank test, and a p‐value less than 0.05 was considered statistically significant.

## 3 RESULTS

The data of 15 patients with boot‐shaped lung cancer were analyzed in this research. The median patient age was 61 years, ranging from 49 to 74 years. The mean volume of PTV was 446.7cc and the median length of the PTV was 22.0 cm (range, 16.2–30.5 cm). The details of the patient characteristics are presented in Table 2.

**TABLE 2:** Treatment planning objectives and dose constraints for the PTV and OARs.

		Clinical constraints	
PTV/OAR	Parameters	Optimal	Variation acceptable
PTV	D_98%_	>57 Gy	>56 Gy
	D_2%_	<66 Gy	<67 Gy
	V_60Gy_	≥95%	≥94%
Lungs	Dmean	≤15 Gy	<16 Gy
	V_5Gy_	≤60%	<65%
	V_20Gy_	≤28%	<30%
	V_30Gy_	≤18%	<20%
Heart	Dmean	≤25 Gy	<28 Gy
	V_30Gy_	≤35%	<40%
	V_40Gy_	≤25%	<30%
Spinal cord	Dmax	≤45 Gy	<48 Gy

In total, 45 treatment plans were generated according to the protocol as described in the Table 2. All plans were normalized to achieve the comparable coverage of PTV (V_60Gy_ = 95%). For dose distribution in the plan target volume, there were statistical differences in almost all parameters between the two different beam alignment techniques. Dual‐isocenter VMAT technique (D‐TV, D‐HV) provided significantly better target dose coverage (HI, CI) than the S‐TV (*p* < 0.05). Compared to the S‐TV plan, both the D‐TV and D‐HV plans also showed a statistically significant increase in the D_98%_ (representing the minimum dose) of the PTV (*p* = 0.03/0.02). However, although jaw‐tracking was applied in D‐TV compared to D‐HV without jaw‐tracking, there were no significant differences in CI or HI (*p* > 0.05). Table 3 shows the comparison of the dosimetric parameters of PTV and OARs for all plans.

**TABLE 3:** Comparison of dosimetric parameters among the three treatment groups.

Parameter	S‐TV	D‐TV	D‐HV	p^a^	p^b^	p^c^
PTV						
D_98%_ (Gy)	57.68 ± 1.14	57.99 ± 1.07	58.08 ± 0.80	0.03	0.02	0.53
D_2%_ (Gy)	65.93 ± 0.95	65.29 ± 0.93	65.37 ± 1.15	0.02	0.06	0.65
D_50%_ (Gy)	63.32 ± 1.06	62.83 ± 1.21	62.66 ± 1.29	0.04	0.23	0.78
Dmean (Gy)	62.96 ± 0.71	62.54 ± 0.60	62.62 ± 0.67	0.04	0.16	0.36
HI	0.13 ± 0.02	0.11 ± 0.02	0.12 ± 0.02	0.00	0.03	0.50
CI	0.75 ± 0.08	0.78 ± 0.08	0.79 ± 0.07	0.01	0.01	0.39
Lungs						
Dmean (Gy)	14.22 ± 1.30	13.84 ± 1.44	14.16 ± 1.42	0.02	0.73	0.02
V_5Gy_ (%)	52.00 ± 7.32	50.78 ± 6.24	53.36 ± 8.48	0.05	0.17	0.01
V_20Gy_ (%)	26.18 ± 2.86	23.72 ± 2.27	24.96 ± 3.09	0.001	0.02	0.004
V_30Gy_ (%)	17.20 ± 1.72	15.69 ± 1.76	16.52 ± 2.07	0.002	0.08	0.05
Heart						
Dmean (Gy)	18.00 ± 7.21	17.70 ± 7.18	17.72 ± 7.18	0.07	0.07	0.78
V_30Gy_ (%)	24.77 ± 11.13	24.22 ± 10.86	23.93 ± 10.83	0.43	0.50	0.73
V_40Gy_ (%)	16.89 ± 8.40	17.29 ± 9.02	16.82 ± 8.95	0.26	0.46	0.69
Spinal cord						
Dmax (Gy)	42.02 ± 2.04	41.25 ± 2.26	41.04 ± 2.12	0.03	0.02	0.73

*Note*: p^a^: S‐TV vs. D‐TV; p^b^: S‐TV vs. H‐TV; p^c^: D‐TV vs. H‐TV.

Abbreviations: CI, conformity index; Dmax, maximum dose; Dmean, mean dose; D‐TV, dual‐isocenter jaw‐tracking VMAT based on TrueBeam; HI, homogeneity index; H‐TV, dual‐isocenter VMAT based on Halcyon linear accelerator.; S‐TV, single‐isocenter jaw‐tracking VMAT based on the Varian TrueBeam linear accelerator.

**FIGURE 2:**
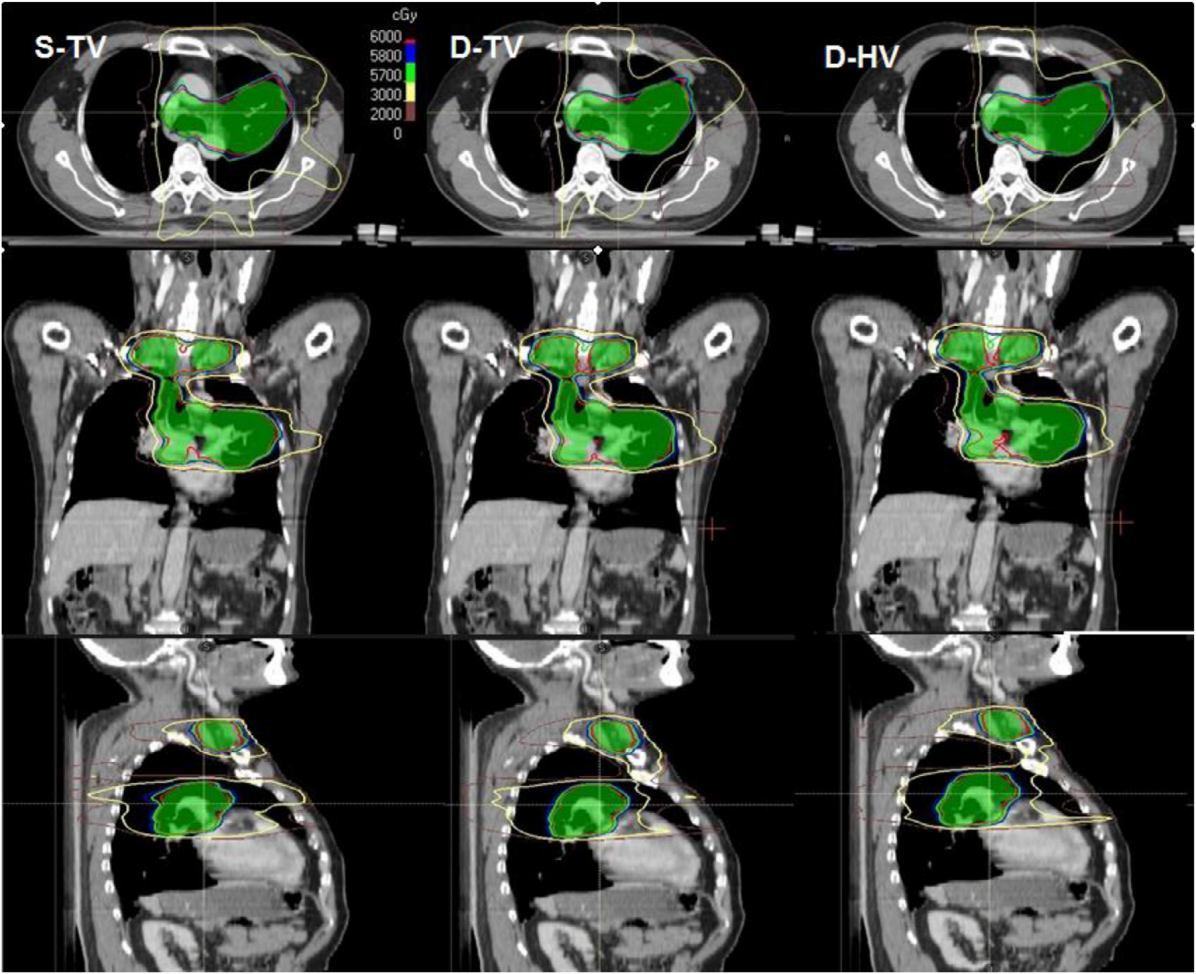
The comparison of dose distribution in S‐TV (left), D‐TV (middle), and D‐HV (right) for one patient with the irradiation isodose curves. D‐TV, dual‐isocenter jaw‐tracking VMAT based on TrueBeam; H‐TV, dual‐isocenter VMAT based on Halcyon linear accelerator, S‐TV, single‐isocenter jaw‐tracking VMAT based on the Varian TrueBeam linear accelerator.

**FIGURE 3:**
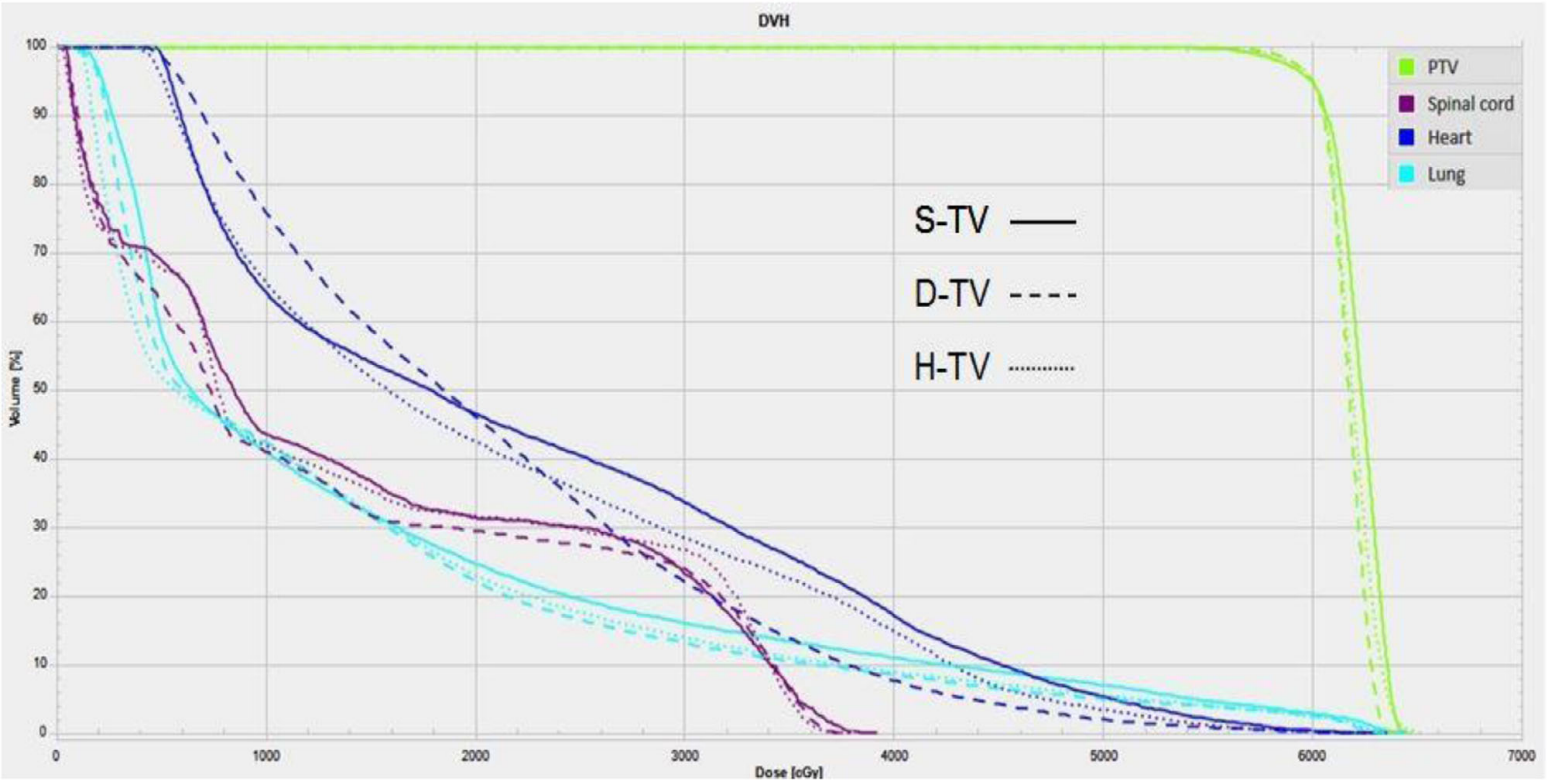
Dose volume histograms (DVHs) comparison between the three treatment groups for an example case.

For the total lung, V_5_, V_20_, V_30_ and MLD values were lower in D‐TV plans than in S‐TV and D‐HV plans, and the majority of these differences were statistically significant. The MLD was 13.84 ± 1.44 Gy (mean ± SD) for D‐TV and 14.22 ± 1.30 Gy and 14.16 ± 1.42 Gy for S‐TV and D‐HV, respectively. The MLD was statistically reduced for D‐TV technique compared to both S‐TV (*p* = 0.02) and D‐HV (*p* = 0.02). Although the mean V_5_ was lower in the D‐TV, there was no statistical difference between S‐TV and D‐TV. However, in three out of fifteen patients compared to S‐TV, the V_5_ value was slightly higher for the D‐TV plan. When comparing D‐TV and D‐HV, V_5_ was significantly lower in D‐TV (50.78 ± 6.24 vs. 53.36 ± 8.48%, *p* = 0.01). Across the entire cohort, the median reduction in lungs‐V_20_ for the D‐TV compared to the S‐TV was 2.1% (range, 0.6%–5.6%), as measured by absolute differences. Meanwhile, one third of the patients had a decrease in V_20_ of more than 3%. Statistically significant differences were found in V_20_ among the three techniques (*p* = 0.001, *p* = 0.02, *p* = 0.004, respectively), as listed in Table 3. Compared to the S‐TV, the median reduction in V_30_ for the D‐TV was 1.6% (range, 0%−4.8%), which was statistically different (*p* = 0.002). The dose distribution and dose‐volume histograms (DVHs) of three VMAT techniques for the representative patient are exhibited in Figures 2 and 3. Apparently, a lower dose delivered to the normal lung was observed in the D‐TV and D‐HV plans, respectively. None of the differences in the dose of the heart were significant (*p* > 0.05). Compared to the S‐TV plan, the maximum dose of the spinal cord was statistically lower in the S‐TV and D‐HV plans (*p* = 0.03, *p* = 0.02, respectively).

The total number of MUs and delivery time for each plan was calculated by using the RayStation 9.0 TPS. In comparison to S‐TV, dual‐isocenter plans statistically increased the MUs (*p* < 0.05), and D‐TV increased the average delivery time from 2.28 to 3.74 min (*p* < 0.05). But, the average delivery time for H‐TV (1.82 ± 0.56 min) was slightly lower than that for S‐TV (2.28 ± 1.22 min), thus not significantly impacting the beam‐on time. However, the actual set‐up and verification of the patient for the second isocenter (iso2) would take additional time, which would extend the treatment delivery. All plans met the gamma criteria of 3%/3 mm. There was no statistical difference in QA pass rates between the three techniques. Full details of the number of MUs, delivery time and QA gamma pass rate percentage difference can be found in Table 4.

**TABLE 4:** The detailed information on total number of MUs, delivery time, and QA pass rates for all 15 patients.

Parameter	S‐TV	D‐TV	D‐HV	p^a^	p^b^	p^c^
MUs	740.49 ± 101.59	807.28 ± 118.77	842.71 ± 147.15	0.001	0.03	0.65
γ pass rates (%)	98.3 ± 1.1	98.1 ± 1.5	98.5 ± 0.8	0.72	0.65	0.78
Delivery Time (min)	2.28 ± 1.22	3.72 ± 1.28	1.80 ± 0.56	0.001	0.11	0.001

*Note*: p^a^: S‐TV vs. D‐TV; p^b^: S‐TV vs. H‐TV; p^c^: D‐TV vs. H‐TV.

Abbreviations: D‐TV, dual‐isocenter jaw‐tracking VMAT based on TrueBeam; H‐TV, dual‐isocenter VMAT based on Halcyon linear accelerator; S‐TV, single‐isocenter jaw‐tracking VMAT based on the Varian TrueBeam linear accelerator.

## 4 DISCUSSION

As previously mentioned, the application of VMAT technique embodies its value in treating complexly shaped tumor targets compared to IMRT. In the present treatment planning study based on 15 locally advanced lung cancer patients with boot‐shaped tumor targets, we demonstrated the potential to improve the target dose distribution (HI and CI) and decrease in normal lung dose while utilizing dual‐isocenter jaw‐tracking VMAT.

The treatment of lung cancer with radiotherapy carries a risk of radiation pneumonitis. Meta‐analysis and several studies have demonstrated that dosimetric parameters, including mean lung dose, lung‐V_5_, V_20_, and V_30_ are related to the development of radiation pneumonitis.^23^, ^24^, ^25^, ^26^ Tsujino et al.^25^ showed that lung‐V_20_ and absolute lung volume received at a dose of 5 Gy (V_S5_) were independent and significant risk factors correlated with severe RP. Barriger et al.^26^ found that MLD was an important predictive factor for RP in non‐small‐cell lung cancer patients. Thus, it is crucial to maintain the lung dose within constraints that can significantly decrease the occurrence of RP. The results of this study showed that for boot‐shaped lung cancer patients, D‐TV technique provided statistically significant better protection for the total lung except for the low dose areas of lungs than S‐TV. On average, V_20_ was significantly (*p* < 0.01) reduced from 26.18% to 23.72% by using the D‐TV technique. Meanwhile, five out of fifteen patients had a decrease in V_20_ of more than 3%. V_30_ was significantly (*p* < 0.01) reduced from 17.20% to 15.69%. The average V_5_ was also found to be lower in the D‐TV compared to the S‐TV, but the difference was not statistically significant. The results are similar to the conclusions drawn in Sun et al.’s study. The researchers investigated the dosimetric variances between single and dual‐isocentric VMAT plans for 11 patients who underwent synchronous bilateral whole‐breast irradiation and found that using dual‐isocentric VMAT plans significantly decreased lung dose metrics such as V_10_, V_20_, V_30_, V_40_, and D_mean_.^19^ Boman et al.^18^ found that V_5_, V_20_, and Dmean of lungs can be significantly reduced in the dual isocenters group for bilateral lymph node positive breast cancer. This suggests that dual‐isocentric solution may be required for very large and complex tumor target. However, Timmeren et al showed that for multiple lung metastases SBRT, the MLD was significantly lower with multi‐isocentric technique compared to single‐isocentric approach, but there were no significant differences in V_20_, and demonstrated that single‐isocenter SBRT did not result in excessive radiation dose to the normal lung tissue.^17^ The reason for this difference may be that lung tumors treated with SBRT are typically small and regular in shape, which may explain the ability to achieve precise dose delivery without the need for multiple isocenters. For the large targets, the multi‐leaf collimators need to travel a greater distance to provide the target coverage to entire tumor, potentially resulting in increased low‐dose radiation exposure to the non‐target tissues such as the normal lungs. It may indicate that radiation treatment planning system can more easily optimize a small and regularly shaped tumor target compared to a large and complex one. If the large target is split into two well‐proportioned targets, each using an isocenter and appropriate beams, and optimized together, better results may be achieved compared to optimizing the large target as a whole. None of the lung parameters were significantly different between S‐TV and D‐HV except for V_20_. D‐TV was statistically significantly more beneficial than D‐HV for lung protection. This is probably due to S‐TV and D‐TV utilizing jaw tracking while Halcyon linear accelerator does not support this feature. Although the dosimetry parameters of the S‐TV and H‐TV met the variation acceptable clinical constraints, they did not meet the more stringent optimal clinical constraints. Among the treatment groups, two out of 15 patients in the D‐TV group had a V_30_>18%, while S‐TV and D‐HV had five and four patients, respectively. V_30_ was one of the best predictor of RP and patients with a V_30_ of 18% or less were associated with a 6% rate of RP compared to 24% in patients with a V30 greater than 18%, as described by Hernando et al.^24^

This study also showed that dual‐isocenter techniques (D‐TV, D‐HV) achieved significantly superior HI and CI when target coverage was uniform (V_60Gy_ = 95%). These results are in line with a recent study that compared the dosimetric evaluation of mono‐ and dual‐isocentric VMAT techniques for non‐contiguous spine SBRT.^27^ The additional coplanar half arcs of iso2 may be beneficial in terms of increasing treatment angles and delivering an ideal PTV dose distribution while minimizing exposure to nearby OARs.

Additionally, a few investigators have reported the feasibility of dual‐isocenter techniques. The dual isocenter method allowed for the treatment of complex patients with improved plan quality and greater accuracy in treatment delivery in the study by Palmiero et al.^28^ In the previous report by Kim et al, Halcyon could use dual isocenters to create complex extended‐field plans and offer efficient and fast treatment delivery.^29^ Chuter et al demonstrated that patients treated for cervical cancer exhibited large inter and intra‐fraction anatomical changes, using the dual isocenter technique could help maintain the required plan quality for inter‐fraction changes.^30^ In this study, the dose verifcation (γ evaluation using the EPID) confirmed that all plans could be reliably delivered. As expected, MUs of dual‐isocenter plans were higher and D‐TV had the longest delivery time. The maximum gantry rotation speed of the Halcyon is 24 degrees per second, while that of the Truebeam is 6 degrees per second, so the delivery time of D‐HV was shortest. As dual isocenters technique was not yet widespread used clinically, the delivery time in actual treatment was not investigated and the positioning verification for the dual‐isocenter would take additional time. The automatic treatment couch movement function can be used to move from the first isocenter to the second, eliminating the need for the radiotherapist to enter the treatment room twice. Additionally, since this is only a treatment planning study, to the best of our knowledge, no clinical outcomes associated with dual‐isocenter technique have been established, future clinical follow‐up is necessary to determine the potential value of dual‐isocenter jaw‐tracking VMAT for boot‐shaped lung cancers.

## 5 CONCLUSION

In summary, the dual‐isocenter jaw‐tracking VMAT has the potential to not only provide better target dose conformity and homogeneity but also reduce the normal lung dose for boot‐shaped lung cancer radiotherapy. This approach may potentially decrease the risk of developing RP at the cost of increased treatment time.

## AUTHOR CONTRIBUTIONS

Conception and design: L.Z. and K.N.‐S. Acquisition of data: L.Z. Analysis of data: F.L.‐D., G.P.‐S. Writing, review and/or revision of the manuscript: L.Z., H.C., F.L.‐D., K.N.‐S., S.M.‐Z., G.P.‐S. All authors reviewed the manuscript.

## CONFLICT OF INTEREST STATEMENT

The authors declare no conflicts of interest.

## ETHICS STATEMENT

Ethical approval for this low‐risk study has been obtained from the local Ethics Committee.

## CONSENT FOR PUBLICATION

Not applicable.

## Data Availability

All data included are available by contacting the corresponding author.
